# A safe and effective vaccine against bovine leukemia virus

**DOI:** 10.3389/fimmu.2022.980514

**Published:** 2022-08-10

**Authors:** Guillermo Suárez Archilla, Gerónimo Gutiérrez, Cecilia Camussone, Luis Calvinho, Alejandro Abdala, Irene Alvarez, Marcos Petersen, Lautaro Franco, Gabriel Destefano, Gustavo Monti, Jean-Rock Jacques, Thomas Joris, Luc Willems, Karina Trono

**Affiliations:** ^1^ Instituto de Investigación de la Cadena Láctea (INTA-CONICET), Estación Experimental Agropecuaria Rafaela, Rafaela, Argentina; ^2^ Instituto de Virología e Innovaciones Tecnológicas, Centro de Investigaciones en Ciencias Veterinarias y Agronómicas, (INTA-CONICET), Hurlingham, Argentina; ^3^ Quantitative Veterinary Epidemiology Group, Wageningen University and Research, Wageningen, Netherlands; ^4^ Molecular and Cellular Epigenetics (GIGA) and Molecular Biology (TERRA), University of Liège (ULiège), Liège, Belgium; ^5^ Molecular and Cellular Epigenetics, Interdisciplinary Cluster for Applied Genoproteomics (GIGA) of University of Liège (ULiège), Liège, Belgium

**Keywords:** BLV, vaccine, attenuated strain, retrovirus, HTLV

## Abstract

Previous attempts to develop a vaccine against bovine leukemia virus (BLV) have not been successful because of inadequate or short-lived stimulation of all immunity components. In this study, we designed an approach based on an attenuated BLV provirus by deleting genes dispensable for infectivity but required for efficient replication. The ability of the vaccine to protect from natural BLV infection was investigated in the context of dairy productive conditions in an endemic region. The attenuated vaccine was tested in a farm in which the prevalence rose from 16.7% in young cattle at the beginning of the study to more than 90% in adult individuals. Sterilizing immunity was obtained in 28 out of 29 vaccinated heifers over a period of 48 months, demonstrating the effectiveness of the vaccine. As indicated by the antiviral antibody titers, the humoral response was slightly reduced compared to wild-type infection. After initial post-vaccination bursts, the proviral loads of the attenuated vaccine remained most frequently undetectable. During the first dairy cycle, proviral DNA was not detected by nested-PCR in milk samples from vaccinated cows. During the second dairy cycle, provirus was sporadically detected in milk of two vaccinated cows. Forty-two calves born from vaccinated cows were negative for proviral DNA but had antiviral antibodies in their peripheral blood. The attenuated strain was not transmitted to sentinels, further supporting the safety of the vaccine. Altogether, these data thus demonstrate that the vaccine against BLV is safe and effective in herd conditions characterized by a very high incidence. This cost-effective approach will thus decrease the prevalence of BLV without modification of production practices. After facing a series of challenges pertaining to effectiveness and biosafety, the vaccine is now available for further large-scale delivery. The different challenges and hurdles that were bypassed may be informative for the development of a vaccine against HTLV-1.

## Introduction

Bovine leukemia virus (BLV) naturally infects cattle, yak, zebu and water buffalo (1). In the bovine species, major symptoms of BLV infection are lymphoma (enzootic bovine leukemia or EBL) and persistent lymphocytosis (PL). Approximately one third of BLV-infected cows will develop PL while tumors affect 5–10% of animals after long latency periods (4–10 years) (2). At the asymptomatic stage, BLV infection is associated with reduced milk production (3), shortened longevity (4) and immune suppression (5). Because no obvious symptoms are observed in most animals, BLV has been neglected in many regions worldwide. BLV prevalence has nevertheless a major economic impact according to recent prediction models (6). BLV is endemic in Argentina and extends worldwide except in occidental Europe, Australia and New Zealand (2). Previous attempts to develop a vaccine inducing a long-term protection (e.g., synthetic peptides, inactivated viruses, cell lysates, viral subunits, recombinant vaccinia virus, DNA vectors) have not been successful (2) because of inadequate or short-lived stimulation of all immunity components (7). Ideally, the optimal vaccine would therefore contain a number of viral factors able to permanently stimulate the immune response.

A different approach was to design an attenuated BLV virus deleting genes dispensable for infectivity but required for efficient replication (2, 7, 8). In this study, we define infectivity as the ability to establish a persistent infection in a host species. Our concept of the approach was to establish a permanent infection with an attenuated strain that impedes wild-type challenge by activating the anti-viral immune response. In fact, deletions and mutations naturally may occur in BLV proviruses, leading to replication-defective or attenuated clones. The challenge was to identify a non-pathogenic BLV strain able to induce a long-lasting immunity. Since the first BLV recombinant obtained by reverse genetics in 1993 (9), this quest took us some time of test-trial cycles and faced a series of failures. We previously identified a recombinant BLV provirus that was infectious and replicated at very low levels, but elicited a vigorous immune response in 13 female cattle (7). This potential BLV vaccine contained deletions and mutations in genes required for infectivity and replication. This attenuated strain conferred long-term protection after the challenge of cattle with a high dose of wild-type virus (7). The safety of the vaccine was supported by the lack of infection of control sentinels over a five-year period in herd conditions. Although the vaccine was not transmitted from cow-to-calf in 12 animals, maternal milk provided anti-BLV passive immunization (7).

In the present study, we evaluated the effectiveness and safety of this candidate vaccine in a productive dairy farm characterized by high rates of BLV prevalence.

## Materials and methods

### Design of the vaccine and production of the inoculum

**Figure 1:**
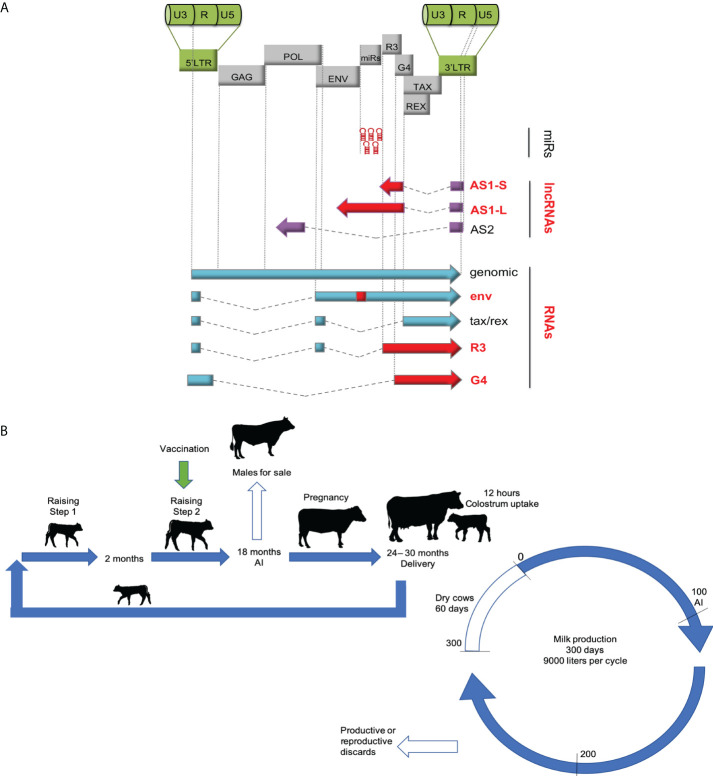
**(A)** The genetic map of the BLV vaccine strain. The pBLV6073DX provirus is isogenic to pBLV344 but contains a mutation in the env gene and deletions in AS1-S, AS1-L, R3 and G4 (red) (9, 10). The viral microRNAs are thus preserved in the vaccine strain. **(B)** Experimental plan of the vaccination trial in the dairy farm. The female calves were vaccinated once at 6 months (+/- 6 months) depending on the drop of anti-BLV antibodies conferred by passive immunity. Standard management practices were then followed in the milk producing herd. Male calves were sold at 18 months while heifers were artificially inseminated (AI). After delivery, the calves suckled colostrum during 12 hours and were then placed in calf individual sticks and bucket fed with bulk-tank milk and balanced food for 60 days (step 1). Then, calves of similar age and weight were moved to pastures (step 2). The cows entered a production cycle during approximately 10 months. The cows underwent an average of 3.5 reproductive cycles.

The BLV vaccine is a recombinant between two previously reported modified strains displaying an attenuated phenotype (10, 11). Briefly, the vaccine strain (pBLV6073DX) has a point mutation in the transmembrane protein gene (T>G at nucleotide 6073) and a partial deletion of the R3-G4 sequences (between positions 6614 and 6997) (**Figure 1A**). This deletion thus also affects the AS1-S and AS1-L antisense transcription. This proviral construct inserted in the pSP64 vector (Promega) was amplified in *Escherichia coli* C3040 (New England Biolabs). The standard protocol for production of the inoculum was based on transfection of a monolayer of CHOK1 cells in 25 cm^2^ cell culture flasks with 2.6 µg of pBLV6073DX and 5.2 µg of linear polyethylenimine (Polysciences) in the presence of 3 ml EMEM (Sigma-Aldrich). Four hours post-transfection, 3 ml of EMEM supplemented with 10% FBS (Internegocios SA) and 3% trehalose were added. Seventy-two hours post-transfection, lysate aliquots (6 ml/dose) were preserved at -80°C in the presence of 15% trehalose.

### Nested-PCR of proviral DNA in blood and milk

Total DNA was extracted from frozen blood or milk using the High Pure PCR Template Preparation kit (Roche), following the manufacturer’s instructions. BLV proviral DNA was amplified by nested-PCR (nPCR) using oligonucleotides external to the deleted region in the vaccine strain (outer forward: 5’-CTCACTTCTGCTTCACCATCC-3’, outer reverse: 5’-GGCAGGCATGTAGAGAGTGG-3’, inner forward: 5’-TGGAAAGAACTAACGCTGACGG-3’, inner reverse: 5’-CCCCAACCAACAACACTTGCTT-3’) as previously described (12). Wild-type and pBLV6073DX proviruses were differentiated by the size of the amplicon migrated on a 2% agarose electrophoresis gel, respectively, 610 and 230 base pairs. Amplification of the bovine 18S gene (adapted from 13) was performed in parallel (forward 18S F: 5’- TTGGATAACTGTGGTAATTCTAGAAGCTAA-3’, reverse 18S R: 5’-CGGGTTGGTTTTGATCTGATAAAT-3’).

### Nucleotide sequencing of the env gene surrounding nucleotide 6073

In addition to nPCR, partial DNA sequencing of the env gene surrounding nucleotide 6073 was performed to validate strain integrity. A thymine or a guanine at nucleotide #6073 indeed attest for a wild-type or a vaccine genotype, respectively. Peripheral blood DNA was amplified by nPCR with primers (env outer forward: 5’- GGTGCGAGAAACCATTCATTC-3’, env outer reverse: 5’-TCGGAGGTTGATGTAATCGG-3’, env inner forward: 5’-AGCCCTTTTTTTGCTCTTCCTG-3’, env inner reverse: 5’- TCTGGTGCAGATGGTAGCAAG-3’). The amplicons were sequenced by capillary electrophoresis with the automatic sequencer GA3500XL (Applied Biosystems) and analyzed by BioEdit software (Freeware Copyright 1991- 2007 Tom Hall).

### Quantification of BLV proviral loads by real time quantitative PCR

The relative BLV proviral loads of wild-type and pBLV6073DX were determined by real time quantitative PCR (qPCR) using oligonucleotides complementary to the pol gene (BLV pol 5F: 5′ CCTCAATTCCCTTTAAACTA-3′; BLV pol 3R: 5′-GTACCGGGAAGACTGGATTA-3′) (12, 14). As previously described, proviral loads were arbitrarily considered as high or low according to an internal calibrator corresponding to 1% of infected cells or 2,400 proviral copies/µg DNA (15). The limit of detection (LOD) of the assay was 5-10 copies/reaction.

### Quantification of anti-BLV immunoglobulins by ELISA

The ELISA assay that quantified the anti-BLV IgGs was described previously (16). Results were expressed as the absorbance at 450 nm normalized to the international E4 OIE reference serum arbitrarily set to 100%. All samples below 25% were stated as negative according to previous validation (16, 17).

### Animal experimentation

Following ethical, welfare and specific requirements for a genetically modified viral strain, the trial was carried out between 2015 and 2021 with the permission of the Argentinean sanitary authority (Expediente SENASA S01-0011591/2014). The farm was a dairy facility that belonged to the Rafaela Experimental Station of INTA (31°11’ S latitude and 61°33’ W longitude) of approximately 300 cows producing milk for human consumption and showing historical endemic rates of BLV infection (**Table S1**).

Animal experimentation was conducted in accordance with the most recent national and international guidelines for animal care and use and following the directive 2010/63/UE of the European parliament. Handling of animals and experimental procedures were reviewed and approved by INTAs Institutional Committee for Care and Use of Experimental Animals (CICUAE‐INTA) under protocol numbers 35/2010 and P18-004.

## Results

### Inoculation of the pBLV6073DX attenuated strain in herd settings

Based on experimental evidence obtained in previous studies carried out under controlled conditions (7, 18), the goal of this study was to evaluate the effectiveness and safety of the candidate vaccine (**Figure 1A**) in a dairy farm characterized by a high rate of BLV prevalence. In this farm, newborn calves were fed with colostrum from their own dams and raised within the herd according to standard productive management measures (**Figure 1B**). Twenty-nine BLV-free calves were inoculated subcutaneously with a single dose (6 ml see M&M) of the vaccine. Vaccination was performed over a period spreading from 4 months to up to one year of age because successful infection with pBLV6073DX strain required low levels of passive anti-BLV antibodies (**Table S2**). Vaccinated animals were mixed with non-inoculated controls according to their birth date. Persistent infection with pBLV6073DX strain was confirmed by nPCR and anti-BLV seropositivity. According to standard practices of the dairy herd, heifers were bred for the first time at 18 months of age. Vaccinated heifers were mixed at all times with similar categories of non-vaccinated cattle and were subjected to the same management practices of the dairy herd. One hundred days post-calving, cows were artificially inseminated. The average parity of the herd was 3.5, delivering approximately one calf per year per cow. The average daily milk production per cow was 25 liters (adjusted at 305 days).

The candidate vaccine was thus evaluated in a commercial dairy farm characterized by a high rate of BLV prevalence. Since the vaccine is an attenuated BLV strain, the main difficulty was to successfully infect the calves in herd settings.

### The pBLV6073DX strain elicited a humoral response in vaccinated cows

**Figure 2:**
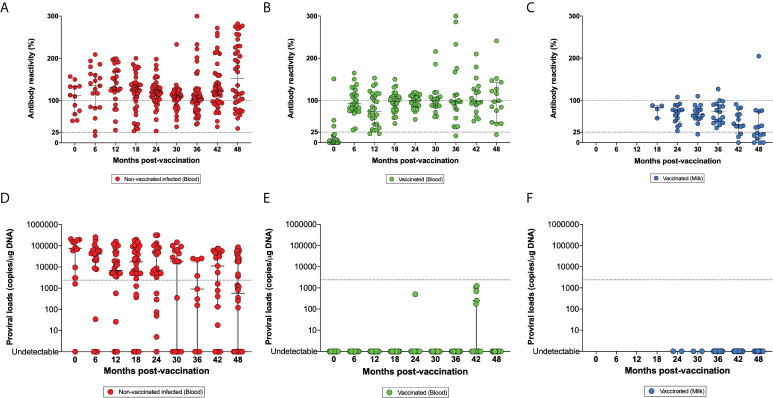
Kinetics of the antibody reactivities **(A–C)** and proviral loads **(D–F)**. Panels **(A, D)** correspond to blood samples of non-vaccinated animals infected with BLV (red). Blood samples (**B, E** in green) and milk (**C, F** in blue) have been collected from vaccinated animals. The dotted lines of panels **(A–C)** indicate the arbitrary cut-off point between high-low (100%) and low-negative (25%) antibody reactivities. In panels **(D–F)**, the dotted line is the threshold separating high and low proviral loads and corresponding to 1% of infected cells or 2,400 proviral copies/µg DNA.

A main risk was that the calves became infected with the wild-type BLV virus before their vaccination with the attenuated strain. Indeed, the overall prevalence of BLV in the herd fluctuated between 84 and 95% between 2015 and 2020 (**Table S1**). BLV transmission requires the transfer of infected cells through iatrogenic procedures (dehorning, milking machines, palpation gloves, needles) and perhaps by hematophagous insects (19). When the trial was initiated, 16.7% of calves were infected with wild-type BLV indicating *in utero* or perinatal viral transmission. The prevalence of wild-type BLV infection gradually increased to 81.1% after the first calving at 27-30 months of age. At the end of the study, only three lactating cows were free of BLV. All the vaccinated cows developed a persistent circulating antibody response against BLV at slightly lower levels compared to wild-type (**Figures 2A, B**).

Vaccinated cows were episodically seronegative; e.g. ID 6484 and ID 107 at 36 and 48m post-vaccination respectively (**Figure 2B**). Anti-BLV antibodies were also present in the milk of the vaccinated cows (**Figure 2C**). Antibody reactivity in the milk decreased with time and progressively reached the cut-off threshold of the ELISA test at approximately 48 months.

These observations thus indicated that the attenuated strain pBLV6073DX stimulated a persistent anti-BLV antibody response (**Figure 2B**).

### The attenuated vaccine replicated at reduced levels

In the dairy herd under study characterized by a high BLV prevalence (84-95%), the proviral loads were heterogeneous but most frequently high (**Figure 2D** **)**. Considering a threshold of 1% of BLV-infected cells corresponding to 2,400 proviral copies/µg DNA (dotted line), wild-type infected animals in the herd carried high proviral loads in their peripheral blood. In contrast, most of vaccinated cows displayed undetectable proviral loads with infrequent sporadic burst of low proviral loads (e.g. at 24 and 42 months) (**Figure 2E**). Consistently, no provirus was amplified in the milk of vaccinated cows considering a detection limit of 5-10 copies/qPCR reaction (**Figure 2F**).

These data thus demonstrate that the candidate vaccine replicated at extremely low levels compared to the wild-type virus.

### The vaccine strain is stable and does not transmit from cow-to-calf

**Figure 3:**
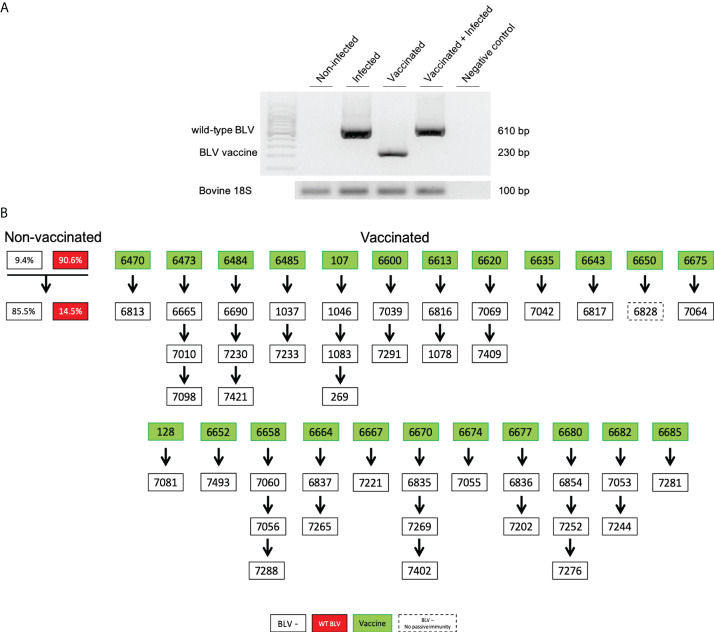
**(A)** Nested-PCR amplicons discriminate animals infected with wild-type BLV, vaccinated (pBLV6073DX) and vaccinated+infected with wild-type BLV. Amplification of the bovine 18S gene is the DNA quality control (13). Note that in the vaccinated+infected lane, only the wild-type amplicon is visible because of the very low proviral loads of the vaccine strain. **(B)** Calves (open rectangles) born from vaccinated dams (green). nPCR and ELISA tests were run during the first week of age. Numbers correspond to their identity ear tags (ID). Calf 6828 did not receive passive immunity (dashed line). As reference, the left panel represents the natural herd prevalence in cows: 90.6% are BLV-infected (red) while 9.4% are negative (empty). These cows delivered 14.5% of calves infected with BLV (red).

To evaluate the possibility of a reversion to a wild-type virus, the animals vaccinated with pBLV6073DX strain were regularly analyzed by DNA sequencing and nPCR. DNAs from peripheral blood samples were sequenced as illustrated in **Figure S3**. The presence of a thymine instead of a guanine at nucleotide #6073 attested for either a reversion, a recombination or a lack of vaccine protection. The absence of reversion or recombination could further be confirmed by nPCR of sequences flanking the R3/G4 deletion (**Figure 3A**). Based on this pattern, regular screening by nPCR demonstrated that the sequence of the vaccine strain was stable over the 4-year trial.

Another important safety parameter is the lack of transmission of the vaccine strain in the environment, in particular to non-infected sentinels. At any time, lack of pBLV6073DX infection of non-vaccinated cows could be evidenced by nPCR (data not shown).

The presence of the pBLV6073DX proviral DNA was also analyzed in milk samples from vaccinated cows. Based on a trimestral sampling schedule, nPCR revealed a sporadic detection of the vaccine strain in milk of two cows (ID 6620 and 6670). This burst was undetectable by real time PCR (**Figure 2F**) and occurred soon after an antibody reactivity peak (**Figures S4, S5**).

The low level of vaccine proviral DNA in the milk was nevertheless insufficient to transmit infection from the cows to their offspring. Indeed, all the calves born from vaccinated cows (n = 42) were stated as negative by nPCR but contained passive anti-BLV antibodies as shown by ELISA (**Figure 3B**). With a single exception (#6828), all calves received anti-BLV passive immunity *via* maternal colostrum. None of these calves born from immunized cows became infected with wild-type BLV (empty rectangles in **Figure 3B**). This lack of transmission sharply contrasted with the high rates of cow-to-calf BLV infection observed in the herd (14.5%, red rectangles; **Figure 3B**).

These data thus show that the pBLV6073DX genome remained stable over a period of at least 4 years. The vaccine strain did transmit neither to sentinels nor to the offspring. It is also noteworthy that, over the study period, clinical manifestations associated with BLV infection (lymphosarcoma) were not observed in vaccinated animals.

### The attenuated vaccine was effective in protecting against wild-type BLV infection

**Figure 4:**
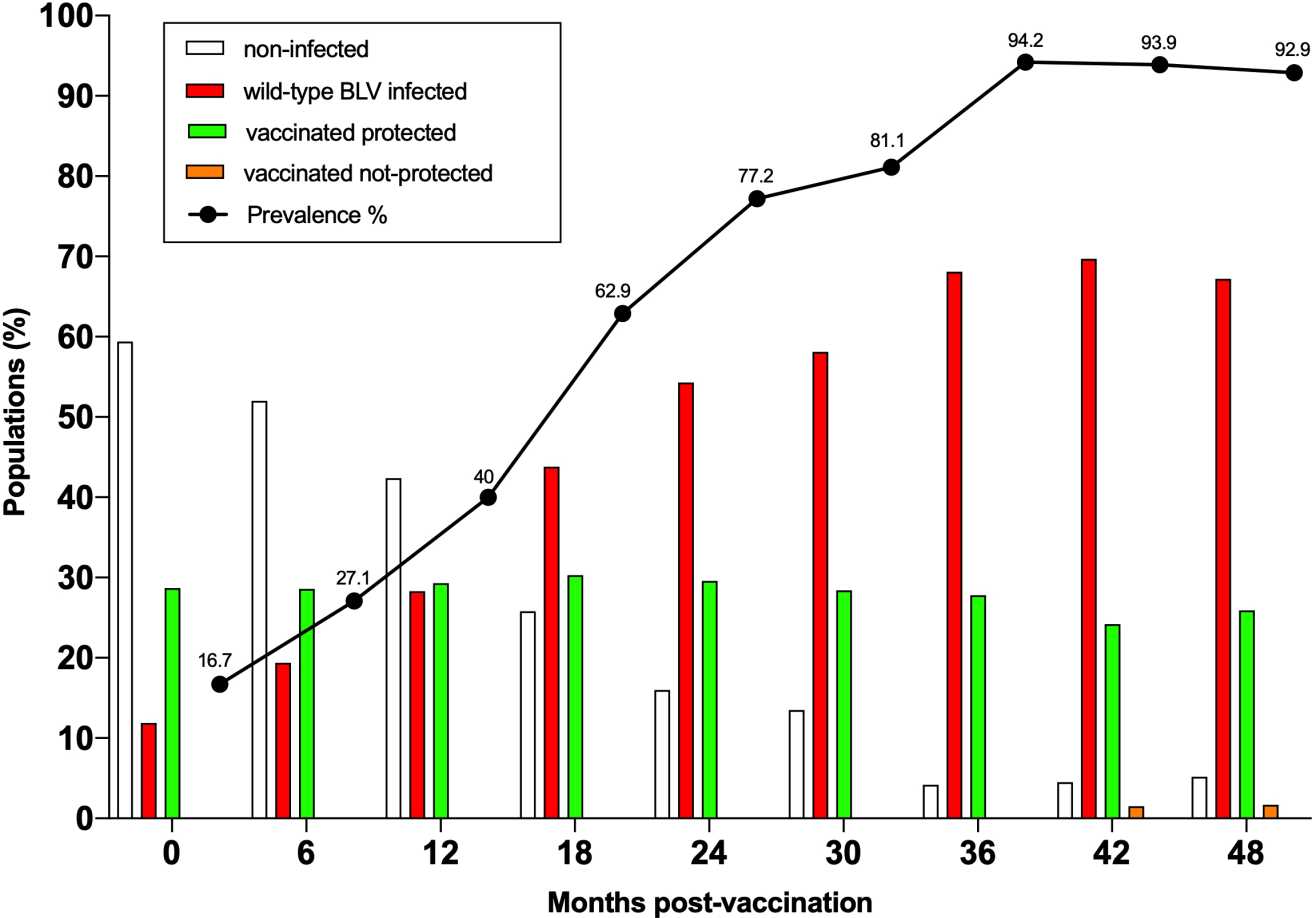
The vaccine is effective despite high incidence of BLV infection. The BLV prevalence indicated by the black line was determined at 6 months intervals from birth up to 4 years of age. The percentages of non-infected and BLV-infected animals are indicated by empty and red bars, respectively. In these conditions, vaccinated animals remained protected from wild-type BLV infection (green). Only one out of 29 vaccinated animals became infected with wild-type BLV at 42 months (vaccinated not-protected in orange).

Based on these safety evidences, the ultimate parameter to be evaluated was effectiveness of the vaccine. Effective protection against wild-type BLV infection was particularly challenging because the herd prevalence grew from 16.7% at the beginning of the trial to >90% after 3 years (**Figure 4**). Consistently, the proportions of BLV-free (empty bars) and BLV-infected (red bars) animals were inversely proportional. In these conditions, the vaccinated cows remained protected from wild-type BLV infection (green bars; **Figure 4**). Among 29 vaccinated animals, only one (ID 6485) became infected by wild-type BLV at 42 months (orange bars). In this particular cow, the vaccine strain could only be sporadically detected by nPCR (**Figure S3**). The proviral loads being particularly low, it is nevertheless expected that cow ID 6485 would not be an effective BLV propagator.

In summary, the attenuated vaccine provided sterilizing immunity in 28 out of 29 animals over a period of 4 years.

## Discussion

In this study, we evaluated the effectiveness and safety of an attenuated BLV strain used as a vaccine in a herd characterized by a very high prevalence of BLV infection (>90% in milking cows between 2015 and 2020). The commercial farm located in Rafaela corresponds to a typical dairy herd in Argentina (15, 20). In these conditions, the attenuated vaccine conferred sterilizing immunity in 28 out of 29 cows over a 4-year period (**Figure 4**). The vaccine was effective against wild-type infection despite lower antibody titers in the peripheral blood likely due to reduced proviral loads (**Figure 2**). In fact, it was relatively unexpected to be able to restrict BLV transmission in the environment of a farm under endemic conditions (**Figure 4**). A major difficulty was to be able to establish an infection with the vaccine strain notably because of passive immunity conferred by the maternal colostrum. There is thus a window of opportunity for the virus to be transmitted prior to vaccination. Since all vaccinated animals remained free of wild-type BLV infection, it appears that passive immunity is particularly effective in young calves. Once the maternal antiviral antibody titers drop around 4-6 months, the rates of BLV transmission accelerate reaching levels > 90% at 3 years (**Figure 4**). Besides the humoral response, it is likely that other mechanisms such as cell-associated immunity mediate the effectiveness of the vaccine. Due to technical limitations associated with experimentation in a farm, it was unfortunately not possible to analyze these other aspects of the host immune response.

Probably the most limiting factor of a live attenuated vaccine is safety. Here, we provided evidence that (i) the proviral loads of pBLV6073DX are extremely low compared to wild-type levels, (ii) the strain does transmit neither to sentinels nor to the offspring and (iii) the vaccine is not pathogenic. Although these conclusions should be validated in large scale vaccination trials, reports from the literature show that cattle infected with low proviral load are not efficient BLV propagators (21, 22). Furthermore, it seems that infection of the attenuated vaccine is abortive because (i) antibody titers decrease with time, (ii) the pBLV6073DX strain can only be detected by very sensitive techniques such as nPCR and (iii) one out 29 vaccinated cows did not resist wild-type infection. All these criteria thus support the safety of the vaccine. Although a period of 4 years is consistent with a mean life expectancy of a cow in a dairy herd, a second vaccine inoculation would be required for older animals.

The pBLV6073DX strain thus displays optimal properties of safety and effectiveness. Of note, this strain which derives from the pBLV344 clone of Belgian origin thus provides protective immunity against the BLV virus strain present in an Argentinean herd. This cross-reactivity is likely associated with the limited diversity of BLV sequences worldwide (23) and/or with the concomitant stimulation of host immunity with multiple viral antigens. A major issue of this approach is that the vaccine was obtained by reverse genetics and is thus a genetically-modified organism (GMO). Although other GMO vaccines are commercially available for bovines worldwide (24), there is a potential risk of recombination of the pBLV6073DX strain. However, reversion to the wild-type 344 sequence is unlikely due to the multiple deletions and mutations. Conceptually, the outcome of this reversion mechanism would be identical to a lack of protection against wild-type infection. It nevertheless remains possible that cellular genes are picked up by the pBLV6073DX strain as observed in other retroviral models (25). This risk is extremely limited because (i) the length of R3/G4/AS1 deletion is small, (ii) the pBLV6073DX strain replicates at extremely low rates and (iii) the BLV does not share similar sequences with the bovine genome. Considering all these arguments and evidence from this study, the Comisión Nacional Asesora de Biotecnología Agropecuaria (CONABIA) and Coordinación de Innovación y Biotecnología of the Ministry of Agriculture from Argentina recently concluded that “the attenuated vaccine is safer for the agroecosystem than the natural BLV from which it derives” (Technical report # 104052660_21). Only well-controlled large-scale vaccination trials will ultimately address this potential risk. Another threat is transmission of BLV to human *via* milk or meat. Although this topic is still controversial (26–29), BLV infection has been associated with breast cancer. In fact, vaccination against BLV should decrease the proviral loads in the milk thereby tempering viral transmission to human. Furthermore, a worldwide reduction of BLV prevalence would also limit the risk, particularly in regions where raw milk is consumed (30, 31). Besides this potential public health issue, the vaccine confers protection against wild-type BLV infection in a herd characterized by a high prevalence. This cost-effective tool may thus reduce BLV prevalence independently of expensive approaches requiring regular testing combined with culling or segregation of infected animals (2, 5).

The current challenge is to make this vaccine available worldwide. This includes large-scale production of doses, their local distribution and approval by the end users. Perhaps the most important risk is the limited interest of the industry that may only focus on short-term profits. This is unfortunately also true for a future HTLV vaccine, a retrovirus closely related to BLV.

In conclusion, the development of a vaccine against BLV faced a series of predicted and unpredicted challenges pertaining to effectiveness and biosafety. These different hurdles are now bypassed and may be useful for the development of other anti-retroviral vaccines such as HTLV-1.

## Author’s note

AA, GS, and LC are researchers from INTA; GD and LF are technicians from INTA; IA, KT, and MP are researchers from INTA and CONICET; CC is a researcher from CONICET; GG and GM are independent researchers; J-RJ is a technician from ULiège; TJ (research fellow) and LW (research director) are members of the FNRS.

## Data availability statement

The original contributions presented in the study are included in the article/**Supplementary Material**. Further inquiries can be directed to the corresponding author.

## Ethics statement

The animal study was reviewed and approved by directive 2010/63/UE European Parliament and protocol numbers 35/2010 and P18-004 INTA’s Institutional Committee for Care and Use of Experimental Animals (CICUAE-INTA). Written informed consent was obtained from the owners for the participation of their animals in this study.

## Author contributions

AA, CC, GSA, and LC managed the animals under study and prepared samples for analysis. CC, GD, GG, GM, GSA, IA, J-RJ, MP, LF, and TJ carried out or contributed to the experiments. KT, LW, AA, and LC planned the study. GG, GSA, KT, and LW analyzed data and drafted the manuscript. All authors contributed to the article and approved the submitted version.

## Funding

This work was supported by the National Institute of Agriculture Technology (INTA), the “Fundación ArgenINTA”, the Asociación Cooperadora de la EEA Rafaela (INTA), the Argentinean National Research council (CONICET), the Belgian National Fund for Scientific Research (FNRS), the Télévie, the NOWALLODOR project of the Walloon government and the “Fonds de maturation” of ULiège Interface.

## Acknowledgments

We want to thank all the workers from the experimental dairy farm of EEA-INTA Rafaela for commitment with the trial in particular Oscar “Cacho” Warnke for his great support and help. We are also grateful to people from the Adventitious laboratory at the Institute of Virology-INTA for running routine assays.

## Conflict of interest

The authors declare that the research was conducted in the absence of any commercial or financial relationships that could be construed as a potential conflict of interest.

## Publisher’s note

All claims expressed in this article are solely those of the authors and do not necessarily represent those of their affiliated organizations, or those of the publisher, the editors and the reviewers. Any product that may be evaluated in this article, or claim that may be made by its manufacturer, is not guaranteed or endorsed by the publisher.
