# Supplementary material for: A safe and effective vaccine against bovine leukemia virus
Source: Front Immunol. 2022 Aug 10;13:980514. doi: 10.3389/fimmu.2022.980514 (PMC9399851; doi:10.3389/fimmu.2022.980514)
Supplement: Supplementary file 1 [file DataSheet_1.docx]

| Year | 2015 | 2016 | 2017 | 2018 | 2019 | 2020 |
| --- | --- | --- | --- | --- | --- | --- |
| BLV Prevalence | 95% | 88% | 92% | 84% | 91% | 94% |

**Supplementary data 1**: **Overall BLV prevalence in the dairy herd between 2015 and 2020**

Blood from all cows in the farm under study was collected by jugular venipuncture. Sera was subjected to anti-BLV IgG ELISA to determine the prevalence of seropositive animals.

|  |  |  |  | BLV status at inoculation | |  |
| --- | --- | --- | --- | --- | --- | --- |
| ID | Status | Number of doses (6 ml/dose) | Route of inoculation | nPCR | ELISA | Age at inoculation (months) |
| 101 | Non-vaccinated | N/A | N/A | - | - | 4.7 |
| 102 | Non-vaccinated | N/A | N/A | - | - | 4.7 |
| 107 | Vaccinated | 1 | subcutanoeous | - | - | 7.1 |
| 110 | Non-vaccinated | N/A | N/A | - | - | 6.3 |
| 112 | Vaccinated | 1 | subcutanoeous | - | - | 6.1 |
| 115 | Non-vaccinated | N/A | N/A | - | - | 5.5 |
| 120 | Non-vaccinated | N/A | N/A | - | - | 7.7 |
| 123 | Non-vaccinated | N/A | N/A | - | - | 7.5 |
| 126 | Vaccinated | 1 | subcutanoeous | - | - | 7.3 |
| 127 | Non-vaccinated | N/A | N/A | - | - | 6.9 |
| 128 | Vaccinated | 1 | subcutanoeous | - | - | 6.7 |
| 130 | Non-vaccinated | N/A | N/A | - | - | 6.6 |
| 131 | Vaccinated | 1 | subcutanoeous | - | - | 6.5 |
| 133 | Non-vaccinated | N/A | N/A | - | - | 6.1 |
| 135 | Non-vaccinated | N/A | N/A | - | - | 5.6 |
| 137 | Non-vaccinated | N/A | N/A | - | - | 5.6 |
| 139 | Non-vaccinated | N/A | N/A | - | - | 8.1 |
| 6457 | Non-vaccinated | N/A | N/A | - | - | 7.3 |
| 6458 | Non-vaccinated | N/A | N/A | + | + | 7.1 |
| 6459 | Non-vaccinated | N/A | N/A | - | - | 7.1 |
| 6460 | Non-vaccinated | N/A | N/A | - | - | 7.0 |
| 6462 | Non-vaccinated | N/A | N/A | - | - | 7.0 |
| 6463 | Non-vaccinated | N/A | N/A | - | - | 7.0 |
| 6464 | Non-vaccinated | N/A | N/A | - | - | 6.9 |
| 6467 | Non-vaccinated | N/A | N/A | - | - | 6.6 |
| 6468 | Non-vaccinated | N/A | N/A | - | - | 6.5 |
| 6469 | Vaccinated | 1 | subcutanoeous | - | - | 6.2 |
| 6470 | Vaccinated | 1 | subcutanoeous | - | - | 6.2 |
| 6471 | Vaccinated | 1 | subcutanoeous | - | - | 6.1 |
| 6472 | Non-vaccinated | N/A | N/A | + | + | 6.0 |
| 6473 | Vaccinated | 1 | subcutanoeous | - | - | 5.9 |
| 6475 | Non-vaccinated | N/A | N/A | - | - | 5.8 |
| 6476 | Non-vaccinated | N/A | N/A | - | - | 5.8 |
| 6477 | Non-vaccinated | N/A | N/A | - | - | 5.8 |
| 6478 | Non-vaccinated | N/A | N/A | - | - | 5.8 |
| 6479 | Non-vaccinated | N/A | N/A | - | - | 5.7 |
| 6481 | Non-vaccinated | N/A | N/A | - | - | 5.7 |
| 6482 | Non-vaccinated | N/A | N/A | - | - | 5.8 |
| 6484 | Vaccinated | 1 | subcutanoeous | - | - | 5.5 |
| 6485 | Vaccinated | 1 | subcutanoeous | - | - | 5.5 |
| 6486 | Non-vaccinated | N/A | N/A | + | + | 5.5 |
| 6488 | Non-vaccinated | N/A | N/A | - | - | 5.2 |
| 6490 | Non-vaccinated | N/A | N/A | - | - | 5.0 |
| 6491 | Non-vaccinated | N/A | N/A | + | + | 5.0 |
| 6492 | Non-vaccinated | N/A | N/A | - | - | 4.8 |
| 6493 | Non-vaccinated | N/A | N/A | - | - | 4.8 |
| 6498 | Non-vaccinated | N/A | N/A | - | - | 7.8 |
| 6499 | Non-vaccinated | N/A | N/A | - | - | 7.6 |
| 6600 | Vaccinated | 1 | subcutanoeous | - | - | 7.4 |
| 6601 | Non-vaccinated | N/A | N/A | + | + | 7.2 |
| 6602 | Non-vaccinated | N/A | N/A | + | + | 7.2 |
| 6603 | Non-vaccinated | N/A | N/A | + | + | 7.2 |
| 6604 | Non-vaccinated | N/A | N/A | - | - | 7.1 |
| 6605 | Non-vaccinated | N/A | N/A | - | - | 7.0 |
| 6607 | Non-vaccinated | N/A | N/A | + | + | 6.7 |
| 6608 | Non-vaccinated | N/A | N/A | - | - | 6.7 |
| 6609 | Non-vaccinated | N/A | N/A | - | - | 6.7 |
| 6612 | Non-vaccinated | N/A | N/A | - | - | 6.7 |
| 6613 | Vaccinated | 1 | subcutanoeous | - | - | 6.7 |
| 6616 | Non-vaccinated | N/A | N/A | - | - | 6.5 |
| 6617 | Non-vaccinated | N/A | N/A | + | + | 6.5 |
| 6619 | Non-vaccinated | N/A | N/A | - | - | 6.4 |
| 6620 | Vaccinated | 1 | subcutanoeous | - | - | 6.3 |
| 6621 | Non-vaccinated | N/A | N/A | - | - | 6.1 |
| 6623 | Non-vaccinated | N/A | N/A | - | - | 6.1 |
| 6626 | Non-vaccinated | N/A | N/A | - | - | 5.9 |
| 6627 | Non-vaccinated | N/A | N/A | - | - | 5.8 |
| 6628 | Non-vaccinated | N/A | N/A | - | - | 5.8 |
| 6635 | Vaccinated | 1 | subcutanoeous | - | - | 14.6 |
| 6636 | Non-vaccinated | N/A | N/A | - | - | 14.6 |
| 6643 | Vaccinated | 1 | subcutanoeous | - | - | 14.3 |
| 6648 | Non-vaccinated | N/A | N/A | - | - | 13.6 |
| 6650 | Vaccinated | 1 | subcutanoeous | - | - | 13.6 |
| 6652 | Vaccinated | 1 | subcutanoeous | - | - | 8.8 |
| 6653 | Non-vaccinated | N/A | N/A | - | - | 8.8 |
| 6654 | Non-vaccinated | N/A | N/A | + | + | 8.6 |
| 6657 | Non-vaccinated | N/A | N/A | - | - | 8.2 |
| 6658 | Vaccinated | 1 | subcutanoeous | - | - | 7.9 |
| 6659 | Non-vaccinated | N/A | N/A | - | - | 7.9 |
| 6662 | Vaccinated | 1 | subcutanoeous | - | - | 7.6 |
| 6663 | Non-vaccinated | N/A | N/A | - | - | 7.5 |
| 6664 | Vaccinated | 1 | subcutanoeous | - | - | 7.2 |
| 6666 | Non-vaccinated | N/A | N/A | - | - | 7.1 |
| 6667 | Vaccinated | 1 | subcutanoeous | - | - | 7.1 |
| 6669 | Non-vaccinated | N/A | N/A | - | - | 7.0 |
| 6670 | Vaccinated | 1 | subcutanoeous | - | - | 7.0 |
| 6671 | Non-vaccinated | N/A | N/A | - | - | 6.9 |
| 6674 | Vaccinated | 1 | subcutanoeous | - | - | 6.5 |
| 6675 | Vaccinated | 1 | subcutanoeous | - | - | 11 |
| 6676 | Non-vaccinated | N/A | N/A | - | - | 6.5 |
| 6677 | Vaccinated | 1 | subcutanoeous | - | - | 6.3 |
| 6679 | Non-vaccinated | N/A | N/A | - | - | 6.2 |
| 6680 | Vaccinated | 1 | subcutanoeous | - | - | 6.1 |
| 6681 | Non-vaccinated | N/A | N/A | - | - | 5.9 |
| 6682 | Vaccinated | 1 | subcutanoeous | - | - | 5.8 |
| 6684 | Non-vaccinated | N/A | N/A | - | - | 5.5 |
| 6685 | Vaccinated | 1 | subcutanoeous | - | - | 5.5 |
| 6687 | Non-vaccinated | N/A | N/A | + | + | 9.4 |
| 6688 | Non-vaccinated | N/A | N/A | - | - | 4.9 |
| 6690 | Non-vaccinated | N/A | N/A | - | - | 4.8 |
| 6691 | Non-vaccinated | N/A | N/A | + | + | 7.7 |

**Supplementary data 2: Protocol of vaccination and BLV status at inoculation**

Blood from calves was collected by jugular venipuncture. BLV proviral DNA was amplified by nPCR and sera was subjected to anti-BLV IgG ELISA to determine the BLV status. Twenty-nine BLV-free calves were inoculated with the vaccine strain. N/A: not applicable


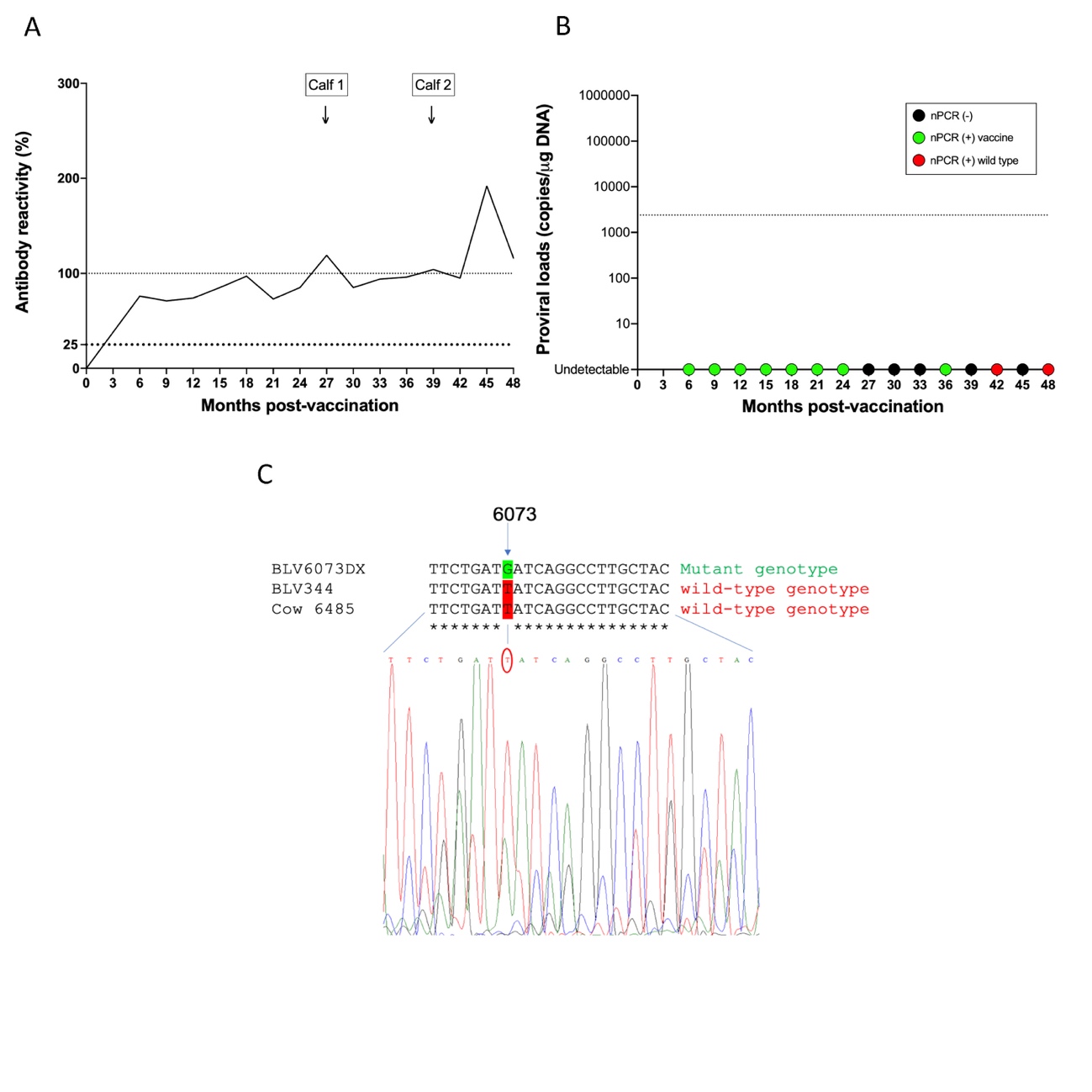
**Supplementary data 3: Vaccinated cow #6485 became infected with wild-type BLV**

Antibody reactivity (A) and proviral loads (B) in the blood of cow #6485. Calves born during the trial are indicated in the upper-left panel. nPCR data are represented as filled circles of different colors indicating a negative result (black), a profile corresponding to the vaccine strain (green) or a lack of protection (red). (C) Nucleotide sequence of the *env* gene surrounding nucleotide 6073. A thymine in position 6073 confirmed wild-type BLV infection instead of a recombination between wild-type BLV and pBLV6073DX.


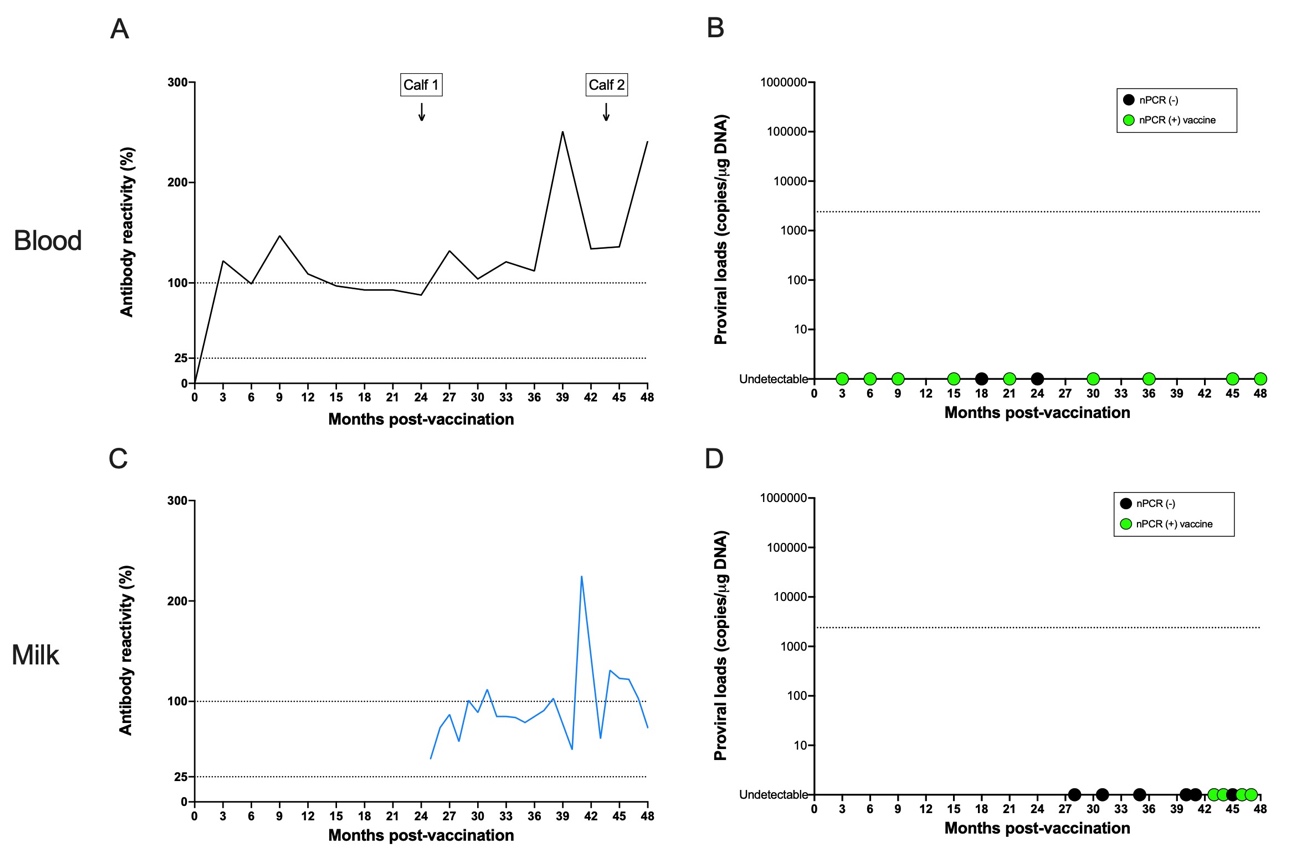


**Supplementary data 4: Identification of pBLV6073DX provirus in the milk from cow #6620**

Antibody reactivity (A and C) and proviral loads (B and D) in the blood (A and B) and milk (C and D). Calves born during the trial are indicated in the upper-left panel. nPCR data are represented as filled circles of different colors indicating a negative result (black) or a profile corresponding to the vaccine strain (green).


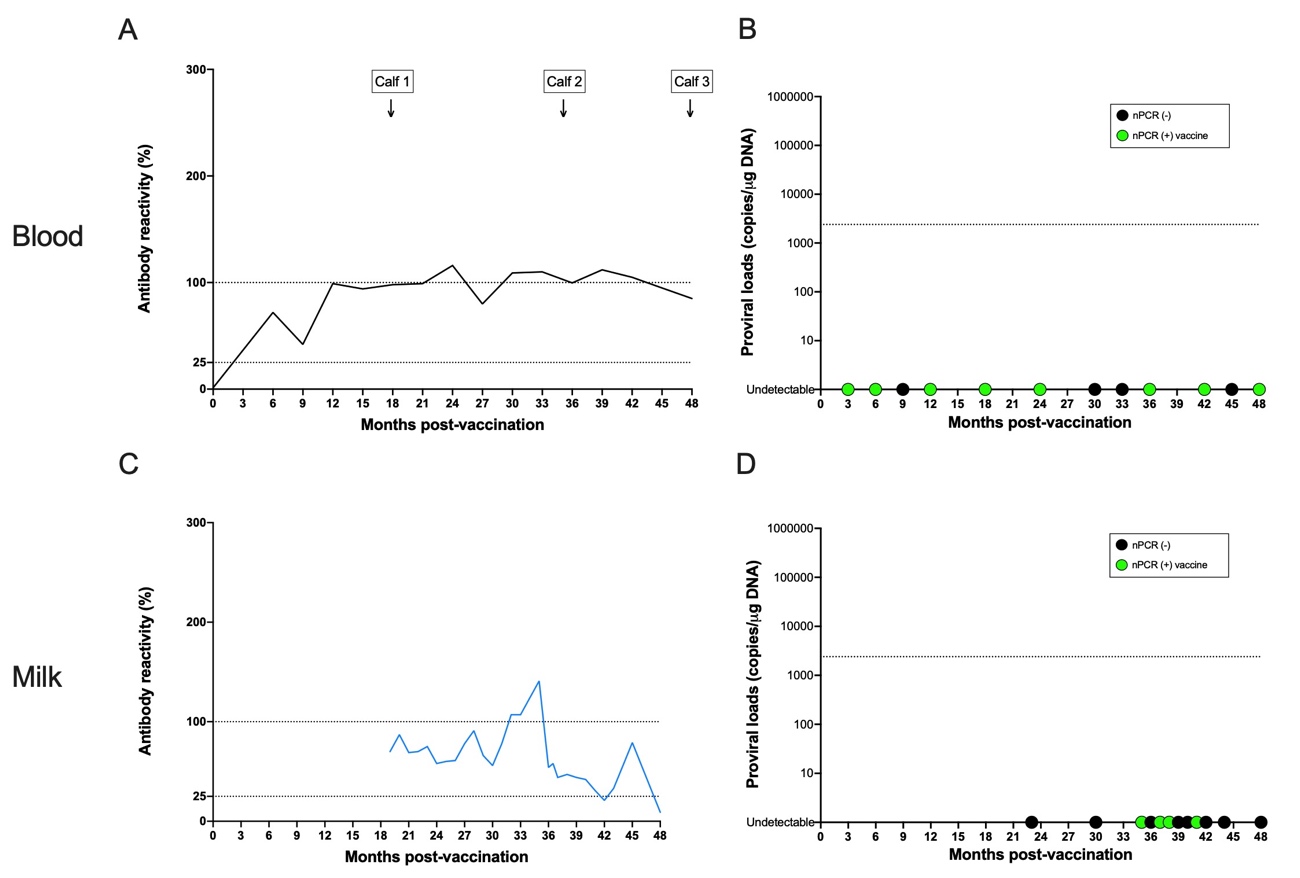


**Supplementary data 5: Identification of pBLV6073DX provirus in the milk from cow #6670**

Antibody reactivity (A and C) and proviral loads (B and D) in the blood (A and B) and milk (C and D). Calves born during the trial are indicated in the upper-left panel. nPCR data are represented as filled circles of different colors indicating a negative result (black) or a profile corresponding to the vaccine strain (green).
